# Response to serotonin reuptake inhibitors in OCD is not influenced by common CYP2D6 polymorphisms

**DOI:** 10.3109/13651500902903016

**Published:** 2009-06-01

**Authors:** Filip C.W. Van Nieuwerburgh, Damiaan A.J.P. Denys, Herman G.M. Westenberg, Dieter L.D. Deforce

**Affiliations:** 1 Laboratory of Pharmaceutical Biotechnology, Ghent University, Ghent, Belgium; 2 AMC, University of Amsterdam, Amsterdam, The Netherlands; 3 UMC, University of Utrecht, Utrecht, The Netherlands

**Keywords:** Obsessive-compulsive disorder, OCD, CYP2D6, venlafaxine, paroxetine, polymorphisms, response, blood levels, plasma levels

## Abstract

The cornerstone of pharmacotherapy for OCD is serotonin reuptake inhibition, either with clomipramine or with selective serotonin reuptake inhibitors (SSRIs). In spite of the success of serotonin reuptake inhibiting drugs, nearly half of OCD patients do not respond to treatment. Treatment response may be affected by genetic polymorphisms of the P450 metabolic system. The four most common enzyme-activity reducing polymorphisms of the P450 CYP2D6 enzyme were determined in 91 outpatients with primary OCD according to DSM-IV criteria, receiving dosages titrated upward to 300 mg/day of venlafaxine or 60 mg/day of paroxetine, using a fixed dosing schedule. Our results show that the investigated CYP2D6 polymorphisms are not a decisive factor in the response to paroxetine and venlafaxine treatment in OCD in spite of their highly significant effect on the blood levels of these medicines.

## Introduction

Serotonin reuptake inhibitors (SRIs) are the most effective pharmacological treatment currently available for patients with obsessive-compulsive disorder (OCD). Still, up to 40–60% of OCD patients do not respond to SRI treatment [1]. We recently reported the pharmacogenetic effect of the 44-bp insertion/deletion 5-HTTLPR polymorphism and the 5-HT2A 1438G/A polymorphism in serotonin-related genes on the efficacy of venlafaxine and paroxetine treatment of OCD [2]. Our findings suggest that these polymorphisms influence the pharmacodynamics of venlafaxine and paroxetine. Polymorphisms in the P450 CYP2D6 enzyme are known to change the pharmacokinetics of venlafaxine and paroxetine [3]. In an effort to characterize whether this pharmacokinetic factor influences the response to paroxetine or venlafaxine treatment, we determined four common polymorphisms of the P450 CYP2D6 enzyme in 91 OCD patients. We investigated the effect of the studied polymorphisms on the blood levels of paroxetine and (*O*-desmethyl)venlafaxine and on the response to paroxetine treatment or venlafaxine treatment.

## Methods

Ninety-one outpatients gave written informed consent for participation in this study, which had been approved by the University of Utrecht Medical Ethical Review committee (Utrecht, The Netherlands). The studied population is described in detail by Denys et al. [2]. Severity of obsessive-compulsive symptoms was rated with the Yale–Brown Obsessive Compulsive Scale (YBOCS), depressive symptoms with the Hamilton Rating scale for Depression (HAM-D) and anxiety with the Hamilton Rating Scale for Anxiety (HAM-A). Only patients with a score of at least 18 on the YBOCS, or at least 12 if only obsessions or only compulsions were present, were included. Patients with a major depressive disorder or patients with a total score of 15 or more on the 17-item HAM-D on admission were excluded. Other exclusion criteria were diagnoses of major depression, bipolar disorder, schizophrenia, other psychotic conditions, substance related disorder within the last 6 months, primary anxiety disorders, personality disorders, use of a concomitant psychotropic drug, organic mental disorders, epilepsy and any structural central nervous system disorder. Furthermore, clinically significant cardiovascular, gastrointestinal, pulmonary, renal, hepatic, endocrine or hematological conditions resulted in exclusion from this study. Of these 91 patients, 17 dropped out of this study because of lack of motivation, uncertain medication use or failure to collect blood for determination of drug plasma concentrations. The 74 remaining patients have following characteristics: 39.2% male; positive family history, 36.1%; age at onset, 19.5 ± 9.0; age at admission to study, 36.3 ± 11.6; baseline YBOCS score, 25.0 ± 5.7.

The patients were randomly assigned in a 12-week, double-blind trial to receive dosages titrated upward to 300 mg/day of venlafaxine or 60 mg/day of paroxetine, using a fixed dosing schedule. (see Denys et al. [4] for details on the dosing schedule). The patients received no additional drugs affecting the P450 enzyme system and were treatment free at baseline for at least 1 month. Primary efficacy was assessed by the change from baseline on the YBOCS, and response was defined as a ≥25% reduction on the YBOCS.

Blood samples were collected from each subject and frozen at −80°C. Blood levels of paroxetine, venlafaxine and *O*-desmethylvenlafaxine were determined at weeks 0, 1, 3, 5, 8, 10 and 12 by high-performance liquid chromatography with fluorescence detection [5]. Blood for the drug level determinations was collected at the response assessment in those weeks, at a random time point after ingestion of the medicine.

DNA was extracted from 10 μl of peripheral blood according to standard procedures. The samples were genotyped using TaqMan® Drug Metabolism Genotyping Assays from Applied Biosystems for the NCBI dbSNP identification numbers rs3892097 (1846G > A polymorphism, resulting in the inactive CYP2D6 allele 4 [6]), rs5030655 (1707T > Del polymorphism, resulting in the inactive CYP2D6 allele 6 [6]), rs1065852 (100C > T polymorphism, resulting in the reduced activity CYP2D6 allele 10 when not in combination with the 1846G > A polymorphism [7]) and rs28371725 (2988G > A polymorphism, resulting in the reduced activity CYP2D6 allele 41 [8]. In a Caucasian population, the frequency of all inactive and all reduced activity alleles is about 35%, of which 80–90% are caused by the four analyzed SNPs [9,10].

## Results

The measured minor allele frequencies for rs3892097, rs5030655, rs1065852, and rs28371725 are 0.24, 0.02, 0.26 and 0.08, respectively. This is in accordance with the minor allele frequencies reported by Applied Biosystems: 0.20, 0.00, 0.21 and 0.11, respectively. Fifty-seven percent of the patients have at least one reduced activity CYP2D6 allele while 13% have two reduced activity CYP2D6 alleles.

To assess the effect of the polymorphisms on the medication plasma levels, all 6 measurements were averaged and two-tailed T-test statistics was performed on this average (see Table I). Paroxetine-treated patients with at least one reduced activity CYP2D6 allele show significantly higher paroxetine levels (*P* = 0.037) compared to patients with normal alleles. Venlafaxine-treated patients with at least one reduced activity CYP2D6 allele show significantly higher venlafaxine levels (*P* = 0.018) compared to patients with normal alleles. When considering the venlafaxine metabolites, insignificant differences are found with lower *O*-desmethylvenlafaxine levels (*P* = 0.397) and higher combined venlafaxine + *O*-desmethylvenlafaxine levels (*P* = 0.065) compared to patients with normal alleles.

**Table I:** Plasma levels and response in patients with at least one reduced activity CYP2D6 allele (poor metabolizers) versus patients with only normal alleles.

	Plasma levels (ng/ml)		Response (YBOCS decrease > 25%)		Response (YBOCS decrease > 35%)	
Paroxetine treated patients (*N* = 35)						
Poor metabolizer (*N* = 17)	177 ± 82	Two-tailed *t*-test *P* = 0.037	75%	χ^2^-test df = 1 *P* = 1.00	55%	χ^2^-test df = 1 *P* = 1.00
Normal metabolizer (*N* = 18)	125 ± 61		80%		50%	
Venlafaxine treated patients (*N* = 39)						
Poor metabolizer (*N* = 22)	390 ± 387	Two-tailed *t*-test *P* = 0.018	52%	χ^2^-test df = 1 *P* = 0.77	44%	χ^2^-test df = 1 *P* = 0.76
Normal metabolizer (*N* = 17)	151 ± 113		58%		53%	

In patients with two reduced activity CYP2D6 alleles, the same results are found but with a higher statistical significance level. The plasma metabolite levels also differ significantly. Patients with two reduced activity CYP2D6 alleles show higher paroxetine levels (*P* = 0.036), higher venlafaxine levels (*P* < 0.0005), lower *O*-desmethylvenlafaxine levels (*P* = 0.017) and higher combined venlafaxine + *O*-desmethylvenlafaxine levels (*P* = 0.007) compared to patients with normal alleles.

In contrast with these findings, the allele frequencies of the individual polymorphisms are almost identical (data not shown) in the responders and non-responders both for the paroxetine group and the venlafaxine group. Pearson chi-square statistics show no significant differences when comparing venlafaxine and paroxetine response in patients with at least one reduced activity CYP2D6 allele *versus* patients with normal alleles (see Table I). Additionally, no significant differences could be found comparing response in patients with two reduced activity CYP2D6 alleles *versus* patients with normal alleles. In the studied population, these Pearson chi-square tests have a post-hoc power to detect an effect size *w* = 0.5 of 0.84.

## Discussion

This is the first study assessing the direct impact of CYP2D6 polymorphisms on pharmacological treatment outcome in OCD. Because CYP2D6 duplications (resulting in ultra-rapid metabolizers) and the non-tested CYP2D6 polymorphisms are too uncommon to be studied in a population of 91 patients, these polymorphisms were not analyzed. Because patient subgroups based on individual genotypes are too small to perform useful statistical tests in the studied population, the patients were divided in groups with no, at least one or at least two reduced activity alleles.

From the results, we conclude that the investigated CYP2D6 polymorphisms are not a decisive factor in the response to paroxetine and venlafaxine treatment in OCD in spite of their highly significant effect on the blood levels of these medicines. Because of a previously reported lack of effect of paroxetine and venlafaxine blood levels on YBOCS response [4], it is expected that the CYP2D6 polymorphisms do not have a major influence on the response. This lack of relationship drug plasma levels and YBOCS response has also been reported for clomipramine, fluoxetine, fluvoxamine and sertraline [11]. On the other hand, a few studies report on a dose–response relationship: A larger study observed higher venlafaxine blood levels in responders compared to non-responders, but found no evidence for a relationship between treatment outcome and blood levels of paroxetine [12]. Hollander et al reported that paroxetine doses of 40 and 60 mg/day (but not 20 mg/day) are effective in treating acute obsessive-compulsive disorder [13]. Yaryura-Tobias et al indicate that doses of venlafaxine less than 225 mg/day are not effective in OCD [14]. Dose–response studies of paroxetine and/or venlafaxine in OCD treatment are scarce. The results in this study show additional evidence that blood concentrations are not a major factor in the response to venlafaxine and paroxetine treatment of OCD.

Given the fact that venlafaxine and paroxetine are metabolized primarily by the CYP2D6 enzymes given the fact that the most common reduced activity alleles do not have a major effect on response and given the fact that other CYP2D6 polymorphisms are rare, previous and future studies on response to venlafaxine and paroxetine treatment in OCD are unlikely to be confounded by CYP2D6 polymorphisms.

## Key points

- The four most common enzyme-activity reducing polymorphisms of the P450 CYP2D6 enzyme were determined in 91 outpatients with primary OCD.
- Patients received dosages titrated upward to 300 mg/day of venlafaxine or 60 mg/day of paroxetine, using a fixed dosing schedule.
- The investigated CYP2D6 polymorphisms are not a decisive factor in the response to treatment in spite of their highly significant effect on the blood levels of these medicines.

## Statement of Interest

The authors do not have any conflict of interest to report regarding this study.
